# The Influence of Sound-Based Interventions on Motor Behavior After Stroke: A Systematic Review

**DOI:** 10.3389/fneur.2019.01141

**Published:** 2019-11-01

**Authors:** Tamaya Van Criekinge, Kristiaan D'Août, Jonathon O'Brien, Eduardo Coutinho

**Affiliations:** ^1^Department of Rehabilitation Sciences and Physiotherapy, Faculty of Medicine and Health Sciences, University of Antwerp, Antwerp, Belgium; ^2^Multidisciplinary Motor Centre Antwerp (M^2^OCEAN), University of Antwerp, Antwerp, Belgium; ^3^Department of Musculoskeletal Biology, University of Liverpool, Liverpool, United Kingdom; ^4^School of Health Science, University of Liverpool, Liverpool, United Kingdom; ^5^Applied Music Research Lab, Department of Music, University of Liverpool, Liverpool, United Kingdom

**Keywords:** sound-based interventions, biomechanics, music, sound, stroke—diagnosis, therapy, stroke rehabilitation

## Abstract

**Objective:** To investigate the effects of sound-based interventions (SBIs) on biomechanical parameters in stroke patients.

**Methods:** PubMed/Medline, Web of Science, the Physiotherapy Evidence Database (PEDro), and the Cochrane Library were searched until September 2019. Studies examining the effect of SBIs on kinematic, kinetic, and electromyographic outcome measures were included. Two independent reviewers performed the screening, and data extraction and risk-of-bias assessment were conducted with the PEDro and Newcastle–Ottawa scale. Disagreements were resolved by a third independent reviewer.

**Results:** Of the 858 studies obtained from all databases, 12 studies and 240 participants met the inclusion and exclusion criteria. Six studies investigated the effect of SBI on upper limb motor tasks, while six examined walking. Concerning quality assessment (Newcastle–Ottawa Quality Assessment Scale and PEDro), the nine cross-sectional studies had a median score of seven, while the randomized controlled trials had a median score of five (fair to good quality). In relation to upper limb motor tasks, only one study found improvements in cortical reorganization and increased central excitability and motor control during reaching after SBI (results of the other five studies were too diverse and lacked quality to substantiate their findings). In relation to walking, results were clearer: SBI led to improvements in knee flexion and gastrocnemius muscle activity.

**Conclusion:** Despite of the heterogeneity of the included studies, evidence was found demonstrating that SBI can induce biomechanical changes in motor behavior during walking in stroke patients. No conclusions could be formulated regarding reaching tasks. Additionally, directions for future research for understanding the underlying mechanism of the clinical improvements after SBI are: (1) using actual music pieces instead of rhythmic sound sequences and (2) examining sub-acute stroke rather than chronic stroke patients.

## Introduction

Stroke is the most common life-threatening neurological disease and the main cause of long term disability in adults (1). After a stroke, only 30% of the survivors have no activity-limitations or participation restrictions (2). The remaining 70% of stroke survivors show persistent impairments in motor function.

It is clear that there is an urgent need for more effective and patient-tailored rehabilitation strategies. Music interventions seem to be beneficial for mobility, upper limb function, and quality of life after stroke (3–6). Results show that music interventions are beneficial for improving clinical outcome measures of both upper and lower extremities and spatiotemporal parameters such as walking speed and step length after stroke (3, 4, 6). Positive effects have also been found on communication-related and quality-of-life outcomes (3). Chen provided an overview of three meta-analyses discussing the effectiveness of music interventions after stroke and highlighted a gap in the literature (5)—the author concluded that although music interventions seem to be promising for enhancing clinical motor recovery after stroke, the quality of evidence is still rather low. It was suggested that more phase I and II studies are necessary as underlying mechanisms, and the nature (true motor recovery or compensation) of these improvements is still unclear (5). Indeed, without taking into account the movement quality of a certain task, it is impossible to discriminate between “true recovery” and “compensation” of motor patterns (7). True recovery would suggest that a patient is able to relearn elemental motor patterns, whereas compensatory strategies mean that an adaptation of remaining motor elements takes place (7). For example, reaching within arm's length can be achieved by either using only the upper limb or using increased sagittal trunk displacements to grasp the object. True recovery of reaching should consist of solely using shoulder flexion and elbow, wrist, and finger extension, while incorporating the trunk to grasp an object should be seen a compensatory strategy. A qualitative biomechanical analysis, assessing joint motion, inter-joint coordination, muscular behavior, or movement synergies, is a required first step in unraveling both concepts (7). Although biomechanical analysis alone cannot fully explain these concepts without an explanation of the underlying neural basis, it is a first step in understanding and explaining possible mechanisms. This additional analysis should be complementary to the extensive research on clinical outcome measures, since biomechanical analysis should always be related to clinical benefits—which have been thoroughly reported (3–6).

Music interventions can be categorized into two types of interventions—passive or receptive (i.e., listening to music) compared to active (e.g., produce music or have an active role during therapy) (8). This study focuses on the effects of passive interventions, as active music interventions, such as playing an instrument, make it difficult to distinguish the cause of the observed changes in motor behavior, which can be due to either the music heard or other behaviors associated with the therapy received. Similar to the review of Tang and Vezeau, the definition of music interventions in this study was extended to include rhythm, since these authors showed that little research has been performed on the role of music listening interventions (MLIs) with stroke patients (8). Hereinafter, we will refer to music listening and rhythmic sound sequence interventions as sound-based interventions (SBIs). It should be noted that interventions that use rhythmic sound sequences are also known as rhythmic auditory stimulation (RAS). Music interventions are able to facilitate and modulate neural plasticity (9, 10), one of the key elements in neurological rehabilitation (11). Activation of the motor regions together with the auditory cortex was seen after SBI, while only the auditory cortex was active before the intervention (9, 12). This suggests that music interventions induce brain reorganization processes and enhance neuronal co-activation and functional coupling of the auditory–motor network after stroke (9, 13, 14). Since music listening is related to several motor–cognitive functions such as memory, attention, semantic processing, and motor function (15), it may be an appropriate tool for neurological rehabilitation. The aims of this study are to investigate the effects of SBI on motor behaviors through a qualitative biomechanical analysis and to discern the underlying mechanisms of clinical improvements (i.e., distinguish between true recovery and compensation). To achieve these aims, we devised a three-part research question:

1. “Are motor behaviors altered *during* SBI?”
2. “Are motor behaviors altered *after* SBI?”
3. “Is SBI *effective* in improving motor behaviors?”

In relation to (1), we will analyze studies that compared similar motor tasks with and without SBI and measured their effects during the delivery of the interventions. Concerning (2), we will look into pre/post-designs that were used to assess the impact of SBI on motor behaviors after the intervention (either immediately after the single intervention or after a longer period of treatment). Finally, regarding (3), we aimed to investigate whether SBI resulted in statistically significant improvements on biomechanical parameters when compared to a control training program. We hypothesize that SBI results in some form of true recovery concerning motor behavior.

## Methods

### Protocol and Registration

This review was conducted according to the Preferred Reporting Items for Systematic Review and Meta-Analysis Statement (PRISMA). The checklist can be found as Appendix S1 (16). The study was registered in the PROSPERO database (no. CRD42018115118).

### Eligibility Criteria

Studies were included if they met the following PICOS criteria:

1. Population: The study population included adults (18 years old or older) diagnosed with ischemic or hemorrhagic stroke (no limitations were set on time since stroke or level of functioning).
2. Intervention: Interventions had to be sound-based, including music listening or listening to rhythmic sequences (MLI or RAS, which could be performed by various instruments, e.g., metronome, synthesizer). At least one group of participants had to perform a task in this condition.
3. Controls: A similar motor act had to be performed without listening to music or rhythmic sequences (control intervention).
4. Outcome: Instrumented 3-D movement analysis with or without electromyography (EMG) had to be performed during a motor task to evaluate the effect of the SBI intervention, and outcome measures had to assess motor function in a biomechanical manner (e.g., movement velocity, movement time, smoothness of motion, joint angles, muscle activity or muscle-related assessment).
5. Study design: All designs except for systematic reviews, meta-analyses, surveys, and case reports.

Studies were excluded using the following criteria:

1. The SBI was not adequately specified (e.g., lack of information concerning music/rhythm, motor task).
2. Studies pertaining to interventions that involved an active music engagement (i.e., singing, playing rhythms on musical instruments).
3. The rhythm was used as a real-time feedback mechanism to aid proprioception and knowledge of performance.
4. Outcome measures assessed by means of a clinical test or motion capture systems were inadequately specified, or only spatiotemporal parameters were investigated since this has been studied thoroughly by previous reviews (3–6).
5. Studies not written in English, Dutch, German, or French.

### Information Sources

A systematic search strategy was conducted using the electronic databases of PubMed, Web of Science, Cochrane, and the Physiotherapy Evidence Database (PEDro). A combination of the following free text words and Medical Subject Headings were used: stroke, cerebrovascular disorder, music, rhythm, rhythmical auditory stimulation, music supported therapy, acoustic, muscle, EMG, kinematics, and biomechanics. The details of the final search strategy, performed in September 2019, can be found in Appendix S2.

### Study Selection

The screening procedure was performed by two independent researchers (TVC and EC). To collect potentially relevant studies, eligibility was screened based on title and abstract based on the provided inclusion and exclusion criteria described above. Full texts were retrieved and evaluated based on the same eligibility criteria. Afterward, full texts were gathered and evaluated on the previously set inclusion criteria. Reference lists were manually screened to identify additional relevant studies. Discrepancies were discussed and resolved with consensus by a third independent person (KD).

### Assessment of Quality

The risk of bias was assessed by two independent reviewers (TVC and KD) by using the Newcastle–Ottawa Quality Assessment Scale (NOS) (17, 18) and the PEDro scale (19). In case of uncertainty at any point during the scoring process, consensus was sought by a third reviewer (JO). The adapted version for cross-sectional studies by Herzog et al. (20) was employed. The NOS is an instrument that assesses the risk of bias by awarding a star for each answer that meets the criteria; a maximum of nine stars can be obtained: four stars for selection, two stars for comparability, and three stars for outcome. Each star given projects a low risk of bias for this criterion. As a criterion for quality, the Agency of Healthcare Research standards were used (21). Included studies were of good quality when they scored three or four stars in the selection domain, one or two in the comparability domain, and two or three in the outcome/exposure domain. Fair quality was assigned to studies that scored two stars in the selection domain, one or two in the comparability domain, and two or three in the outcome/exposure domain. Poor quality was assigned to studies that received zero or one star in the selection domain, zero stars in the comparability domain, and zero or one star in the outcome/exposure domain. For randomized controlled trials (RCTs), the PEDro scale was used, which assesses 11 items such as random allocation of the subjects, concealed allocation, and blinding of therapists and assessors (22). The total PEDro score was considered of good quality when it was six or higher (23).

### Data Extraction and Analysis

Extracted data consisted of subject characteristics (age, gender, time post-stroke, inclusion criteria), outcome measures, motion capture, and sound apparatus (see Table 1). Results were mostly described as a comparison between performing a motor task with and without SBI or, in the case of an RCT study, as a difference between groups based on intervention (see Table 2). Furthermore, a general conclusion per study is also provided.

**Table 1:** Methodology table of the included studies.

**Study**	**Design**	**Participants**				**Sound apparatus**	**MoCap systems**	**Outcome measures**
		**N (ml/f)**	**Age mean (SD)**	**TPS mean (SD)**	**Inclusion**			
Aluru et al. (24)	CS	20 (12/8)	51.6 Y (11.2) Y (19.3) 55.9 Y (12.5)	62.2 M (39.9) 53.8 M (23.9) 65.6 M (58.8)	15°/p/ wrist extension, 5°/a/ wrist extension, no hearing deficits	Metronome, songs	* Electromagnetic motion sensors: trakSTAR, * The Motion Monitor * Bipolar surface electrodes: Delsys	* Amplitude of wrist extension * ECRL and FCU activity: max amplitude and co-contraction, RMS
Ford et al. (25)	CS	11 (10/1)	14–78 Y	>1 Y	Walk independently at 0.63 m/s, no perceptual deficits, no complicating medical history, sufficient motivation, treadmill walking	Metronome	* Optotrak 3020 System	* Coordination: point estimates of relative phase between ipsi-/contralateral limbs and thorax/pelvis * Power: power spectral density, relative power index
Kim et al. (26)	RCT	15 (7/8)	60.07 Y (11.93)	19.40 M (19.49)	Walk 10 m with or without cane, proper communication skills, MMSE >24, VMIQ <3	Metronome	* LUKOtronic AS 202 * Four-channel portable system: QEMG-4 System of Laxtha * Telescan 2.89 software	* Kinematics: hip, knee, and ankle joints * Quadriceps, hamstrings, tibialis anterior, and gastrocnemius activity
Kim et al. (27)	CS	16 (9/7)	47.5 Y (17.65)	26.68 M (27.52)	Brunnstorm arm recovery stage <5, normal hearing, no visual field deficits/neglect, no balance problems, understand instructions	Metronome	* Zebris CMS 10 * WinArm and 3DAwin 1.02 software * Biomonitor ME6000 EMG system * MegaWin 3.1 software	* Movement time * Movement range: max elbow extension * Smoothness: number of movement units * Triceps/biceps brachii activity: %MVIC * Co-contraction ratio: RMS
Mainka et al. (28)	RCT	35 (26/9)	63.7 Y (8.8) 65.5 Y (8.5) 61.1 Y (8.6)	42.6 D (30.1) 46.9 D (23.3) 36.0 D (16.7)	MRC strength <1 for at least one lower limb muscle group, unsafe walking pattern, walk independently with aid for 3 min, no cognitive/language/ psychological disorders	Software Cubase 3 SE (synthesizer), MP3 player	* Force platform, SATEL	* COP sway length * COP sway area * COP mean lateral displacements
Malcolm et al. (29)	Pre/post	5 (5/0)	72.8 Y (6.5)	0.79 Y (0.48)	10° /a/ finger extension, 20° /a/ wrist extension, follow instructions, fair endurance, /p/ ROM at least half of the normal range, MMSE >24	Metronome	* 3-D kinematic analysis, no further specifications of software or hardware used	* Movement time * Movement velocity * Trunk, shoulder, elbow, and kinematic motion (flexion/extension)
Prassas et al. (30)	CS	8 (7/1)	69.6 Y (11)	7.75 M (6.77)	Hemiparetic gait pattern	Synthesizer, sequencer	* Video camera * Panasonic JAVES switcher * Ariel Performance Analysis system * EMG: ASYST software	* ROM hip and knee * Trunk angle and pelvic tilt * CoM: horizontal velocity, vertical and lateral displacements
Sethi et al. (31)	CS	10 (9/1)	67 Y (8.9)	53.3 M (50.9)	>10° extension fingers, >30° elevation in shoulder, >45° /a/ lbow extension, follow two-step commands, no history of other neurological disorders or medical illness	Metronome	* Vicon 612/T40 (plug-in-UE marker set) * SIMM 4.2	* Approximate entropy (variability) of joint motion * Variability error * Peak velocity
Shahine and Shafshak (32)	RCT	76 (40/36)	61.4 Y (5.52) 62.7 Y (3.1)	31.5 M (21.6) 35.6 M (19.5)	Follow instructions, no previous experience with BATRAC, FMA-UE: 26–50	BATRAC	* Nihon Kohden Neuropack 2 * Magstimauditory 200 single pulse stimulator	* Motor-evoked potentials of APB: threshold intensity, max peak-to-peak amplitude, conduction time
Shin et al. (33)	Pre/post	11 (7/4)	44.27 Y (7.04)	3.58 Y (2.22)	No hearing deficit, able to walk independently for at least 10 m, understand commands	Metronome, keyboard	* Vicon Nexus ver. 1.8.5 * Polygon software * ver. 3.5.1	* Kinematic data form the pelvis, hip, knee, ankle, and foot: joint ROM at initial contact, minimal and maximal joint angle during whole cycle
Thaut et al. (34)	CS (long)	10 (8/2)	70.4 Y (10.4)	4 M	Significant gait motor deficits	Synthesizer, music tapes	* EMG: ASYST software	* Gastrocnemius activity: amplitude
Thaut et al. (35)	RCT	20 (10/10)	73 Y (7) 72 Y (8)	16.1 D (4) 15.7 D (4)	Significant gait motor deficits	Metronome, synthesizer	* EMG: ASYST software	* Gastrocnemius activity: variability, RMS
Thaut et al. (36)	CS	21 (13/8)	52.7 Y (13.7)	11.4 M (52)	No neglect, attentional, speech, or sensory deficits	Metronome	* SELSPOT	* Movement time and peak acceleration * Wrist trajectory and velocity * Elbow and shoulder kinematic motion * Variability (CoV)

*SD, standard deviation; MoCap, motion capture; RCT, randomized controlled trial; CS, cross-sectional; long, longitudinal; ml, male; f, female; Y, years; M, months; D, days; TPS, time post-stroke; /p/, passive; /a/, active; °, degrees; m, meter; s, seconds; MMSE, Mini-Mental State Examination; VMIQ, Vividness of Movement Imagery Questionnaire; min, minutes; MRC, Medical Research Council Scale for Muscle Strength; ROM, range of motion; BI, Barthel Index; BATRAC, bilateral arm training with rhythmic auditory cueing; FMA-UE, Fügl-Meyer Upper Assessment Upper Extremity; EMG, electromyography; ECRL, extensor carpi radialis longus; FCU, flexor carpi ulnaris; RMS, root mean square; %, percentage; MVIC, maximum voluntary isometric contraction; CoM, center of mass; CoV, coefficient of variability; COP, center of pressure; SIMM, software for interactive musculoskeletal modeling; APB, abductor pollicis brevis*.

Table 2Synthesis of the results of the included studies.

**Table:** 

**Study**	**Categories of intervention/music conditions**				**Motor task**	**Results**			**Conclusions after RAS**
Aluru et al. (24)	**I1:**Without auditory cue	**I2:**Baby's laughter (happy sounds)	**I3:**Self-selected songs	**I4:**Metronome beat	Bimanual and unimanual wrist flexion/extension	* Cluster analysis divided patients into three groups. * Positive slope (b) quantifies the rate of learning (*b* > 0: increased performance under auditory cues).			RAS is effective in adults with spastic paresis. RAS is not effective in adults with spastic co-contractions. Effectiveness of RAS is unclear in adults with minimal paresis.
						Spastic paresis: * *Metronome beat* -Wrist ext: *b =* 0.86, *p =* 0.03 - FCU act: *b =* 0.0021, *p* < 0.0001 - Co-act: *b =* 0.07, *p =* 0.004 * Self-selected songs: - FCU act: *b =* 0.0010, *p =* 0.04	**Spastic co-contraction:** Without cue: * Wrist ext: *b =* 1.83, **p* <* 0.0001 - ECRL act: *b =* 0.004, *p =* 0.0002 - Co-act: *b =* −0.1, *p =* 0.0006 * * Self-selected songs: - Co-act: *b =* 0.059, *p =* 0.04	Minimal paresis * *Happy sounds:* -Wrist ext: *b =* −0.86, *p =* 0.03 * *Metronome:* -ECRL act: *D =* 0.0022, *p =* 0.02 * *Without:*-FCU act: *b =* 0.0012, *p =* 0.015	
Ford et al. (25)	**I1:** Constant speed (0.63 m/s) + step to beat		**I2:** Constant speed (0.63 m/s) + move arms/legs to beat		Walking on treadmill (30 s acclimation)	* Moving arms/legs to beat resulted in (compared to step to beat):			Moving the arms (1.8 Hz) led to greater arm swing, thoracic and pelvic rotation (out-of-phase rotation).
	Metronome frequency: 1 → 2.2 → 1 Hz (increments 0.2 Hz, 30 s interval)					**Arm/leg motion:** -Improvements ØA_i_, ØA_ni_ (*p <* 0.07) -Greater increases MPA_i_L_i_ and MPA_ni_L_ni_ - Stronger synchronization RPI_ni_		**Trunk motion:** -Greater Ø_P_, Ø_T_, and Ø_PT_ -Greater MPPT	
Kim et al. (26)	**I1:** Visual LMI	**I2:** Kinesthetic LMI (incorporated in analysis)	**I3:** Visual LMI + cue	**I4:** Kinesthetic LMI + cue(incorporated in analysis)	Walking	Differences *pre—post—follow-up* (1 h) of kinesthetic LMI with/without cue			Incorporating auditory step rhythm into locomotor imagery training, improved values in RMS-EMG/kinematic data of affected lower limb muscles during swing and stance phases.
	Each intervention for every participant: 4 days, <15 min					**EMG (RMS**, μV) * Hamstrings: 25.70 (10.04) – 61.89 (27.05) – 45.99 (20.24)/25.70 (12.26) – 47.49 (28.41) – 36.27 (19.39), *F =* 4.008, *p <* 0.05 * Gastrocnemius: 27.68 (10.78) – 42.03 (16.10) – 34.92 (14.07)/26.80 (11.06) – 49.40 (15.14) −35.17 (14.29), *F =* 10.567, *p <* 0.05		**Joint angular displacements (°):** * Knee: 30.65 (7.99) – 42.57 (8.16) – 36.44 (8.74), *F =* 7.723, *p <* 0.05/30.93 (7.36) – 38.57 (7.81) – 35.41 (8.38), *F =* 3.580, *p <* 0.05 * Ankle: 22.41 (3.87) – 29.98 (3.66) – 25.94 (3.89), *F =* 14.823, *p <* 0.05/22.34 (4.04) – 28.52 (5.23) – 25.36 (4.85), *F =* 6.396, *p <* 0.05	
Kim et al. (27)	**I1:** Forward reach with RAS		**I2:** Forward reach without RAS		Forward reaching	Change scores with–without RAS: * Movement time (ms): −108.25 (112.51), *t =* −3.85, *p =* 0.002 * Movement smoothness (MU): −2.96 (2.78), *t =* −4.26, *p =* 0.001 * Elbow extension ROM (°): 4.93 (5.00), *t =* 3.95, *p =* 0.001 * Triceps brachii (%MVIC): 2.14 (3.41), *t =* 2.51, *p =* 0.024 * Biceps brachii (%MVIC): 0.05 (1.76), *t =* 0.11, *p =* 0.911 * Co-contraction ratio: −0.20 (0.28), *t =* 2.75, *p =* 0.015			Improved quality of movement and motor control (decreased movement time and co-contraction ration, increased smoothness, elbow extension ROM, and muscle activation of triceps brachii of the affected arm).
	1 min of reaching (affected arm), 3 min of rest								
Mainka et al. (28)	**I1:** RAS–treadmill training	**I2:** Treadmill training	**I3:** NDT		Standing balance	*ANOVA-RM time effect for length of COP, p = 0.048* *Differences pre–post:*			
	5x/week, 4 weeks					RAS–treadmill training: * Lateral COP (mm) 11.2 (9.5) - 11.6 (9.3), p > 0.05, D = 0.05 * Length of COP (mm): 714.2 (393.5) – 702.5 (525.0), p > 0.05, D = 0.03 * *Sway area COP (mm^2^): 485.6 (602.9) – 397.8 (364.9), p > 0.05, D = 0.18*	Treadmill training: * Lateral COP (mm): 15.9 (10.7) – 13.4 (10.6), p > 0.05, D = 0.23 * *Length of COP (mm):* 938.6 (486.5) – 834.9 (410.9), p > 0.05, D = 0.23 * *Sway area COP (mm^2^): 450.1 (245.1) – 351.5 (181.7), p > 0.05, D = 0.48*	NDT: * Lateral COP (mm): 15.3 (9.9) – 13.0 (10.5), p > 0.05, D = 0.23 * Length of COP (mm): 722.6 (274.7) – 632.6 (147.5), p > 0.05, D = 0.41 * *Sway area COP (mm^2^): 326.6 (216.3) – 259.9 (147.5), p > 0.05, D = 0.36*	No significant differences between groups for COP measurements.
Malcolm et al. (29)	2-week RAS program that provided variation in target rate (cue frequency), reaching excursion, distance, and pattern (1 h on site/2 h home based)				Target reaching	Pre–post * Trunk movements (cm): 8.6 (4.6) – 6.22 (2.34), *t =* 3.23, *p =* 0.002, *D* = 1.1 * Shoulder flexion (cm): 12.54 (6.47) – 15.25 (5.22), *t =* −3.49, *p =* 0.001, *D* = 0.5 * Elbow extension (cm): 6.81 (5.7) – 7.8 (4.6), *t =* −0.82, *p =* 0.21 * Movement time (s): 8.08 (3.1) – 6.22 (1.9), *t =* 2.78, *p =* 0.0245, *D* = 0.75 * Reaching velocity (cm/s): 35.2 (28.1) – 42.2 (24.9), *t =* −2.18, *p =* 0.021, *D* = 0.3			Participants demonstrated substantial decreases in compensatory reaching movement.
Prassas et al. (30)	**I1:** Walk with rhythm		**I2:** Walk without rhythm		Walking	Rhythm–no rhythm: * Trunk angle (°): 96 – 96, *F* < 1, *p >* 0.05 * CoM hor velocity (m/s): 0.6 – 0.6, *F* < 1, *p >* 0.05 * Vert CoM (cm): 2.8 – 3.3, *F =* 5.32, *p =* 0.032 * Hor CoM (cm): 10.1 – 11, *F* < 1, *p >* 0.05 * Pelvic tilt (°): 180 – 181, *F* < 1, *p >* 0.05 * Knee ROM p: 47 – 47, *t =* 0.03, *p >* 0.05 * Knee ROM np: 55 – 56, *F* < 1, *p >* 0.05 * Hip ROM p: 30 – 29, *F =* 1.92, *p >* 0.05 * Hip ROM np: 33 – 36, *F =* 2.02, *p >* 0.05		Paretic–non-paretic (symmetry): * Knee ROM no rhythm: 47 – 56, *t =* −3.661, *p =* 0.001 * Knee ROM rhythm: 47 – 55, *t =* −3.343, *p =* 0.003 * Hip ROM no rhythm: 25 – 36, *t =* −3.84, *p =* 0.001 * Hip ROM rhythm: 30 – 33, *t =* −1.593, *p =* 0.126	Hip joint ROM of the affected/non-affected sides became more symmetrical.CoM vertical displacement decreased, indicating improvement in mechanical efficiency.
	3x over a 5-week period, interval 2 weeks, walking−3 min rest—practiced 1 min tapping to rhythm of music—walking								
Sethi et al. (31)	**I1:** Fast speed	**I2:** Rhythm		**I3:** Self-selected speed	Forward target reaching	Fast > self-selected * Peak velocity (*z* = −3.18): 0.80>0.50, *p =* 0.002	Rhythm > self-selected * Variability: (*z* = −3.18) - Shoulder: *p <* 0.016 - Elbow: *p <* 0.05 - Wrist: *p <* 0.012 - PIP: *z* = −2.51, *p <* 0.025 * Peak velocity (*z =* −2.41): 0.67>0.50, *p <* 0.01 * Variable error: *p =* 0.80	Fast > self-selected * Variability (*z =* −3.18): - Shoulder: *p <* 0.012 - Elbow: *p <* 0.025 - PIP: *p >* 0.05 * Variable error: *p =* 0.50	Reaching at fast speed/cues alters the temporal structure of variability, without compromising the accuracy of the reaching movements.
Shahine and Shafshak (32)	**I1:** BATRAC (5 min in phase, 10 min rest, 5 min anti-phase, 10 min rest, repeat—total of 20 min /a/ training)		**I2:** Unilateral UE training (ROM, strengthening, fine motor tasks—equivalent intensity)		Forward and backward reach	Motor-evoked potential paretic abductor pollicis brevis: 1)Time x group: all parameters *p =* 0.001 2) Time (pre–post)			BATRAC induced significant changes in MEP parameters, suggesting better cortical reorganization and/or increased central excitability (central neurophysiological effects).
	1 h, 3x/week, 8 weeks (24 h)					BATRAC: * MEP rest threshold (%): 85.7 (11.5) – 79.7 (12.3), *p =* 0.001 * CM conduction time (ms): 12 (2.4) – 10.9 (2.6), *p =* 0.003 * MEP amplitude ratio: 0.09 (0.11) – 0.14 (0.11), *p =* 0.001		Unilateral: * MEP rest threshold (%): 83.4 (16.1) – 82.8 (15.1), *p =* 0.10 * CM conduction time (ms): 10.7(2.3) – 10.6 (1.1), *p =* 0.10 * MEP amplitude ratio: 0.13 (0.12) – 0.13 (0.15), *p =* 0.16	
Shin et al. (33)	Walking with RAS: 30 min, 3x/week, 4 weeks a)10 m walk, 3x without RAS, self-selected walking speed b) Walking cadence was calculated c) Initial tempo with metronome d) RAS with keyboard e)Continue cue of 2 min, walking with finger tap for 1 min f) Walking: 3–6x, 10 m, rest 1–3 min, 5–8 repetitions g) 1–2 min fading out				Walking	Only significant differences between pre–post:			Gait training with RAS has beneficial effects for kinematic patterns in patients with hemiplegia. Sub-acute stroke patients were shown to have significant increases in GDI score, suggesting that sub-acute patients are more likely to respond to RAS than chronic patients.
						All stroke: * Max knee flexion in mid-swing: 48.88 (4.31) – 55.31 (3.90), *p =* 0.021 * Maximal ankle DF in terminal stance: 13.79 (1.27) – 16.1 (1.42), *p =* 0.026	Sub-acute stroke: * Maximal knee flexion in mid-swing: 45.15 (3.59) – 56.42 (4.74), *p =* 0.043 * GDI: 80.88 (3.82) – 88.99 (5), *p <* 0.05	Chronic stroke: * External/internal hip rotation at IC: 0.26 (3.57) −−3.98 (3.13), *p =* 0.028 * Maximal ankle DF in terminal stance: 14.08 (1.89) – 16.85 (1.72), *p =* 0.028 * External/internal foot rotation at IC: −6.23 (2.47) −−9.69 (1.44), *p =* 0.028 * Maximal internal foot rotation at push-off: 0.60 (3.19) – −5.77 (2.02), *p =* 0.028	
Thaut et al. (34)	**I1:** With rhythm: musical composition in renaissance style		**I2:** Without rhythm		Walking	**Mean % change of gastrocnemius activity between with and without rhythm:**Time 1 – Time 2 – Time 3 – overall change (significance) * Increase in amplitude (μV/ms) during push-off: -Affected: 4.8 – 11.4 – 16.3 – 10.8, *p <* 0.05 - Non-affected: −7.7 – −8.3 – −9.6 – −8.5, *p >* 0.05 * Decrease in amplitude variation (μV/m) affected side: 14.9 – 15.1 – 19.5 – 16.5, *p <* 0.05* Decrease in amplitude (μV/m) during swing phase: 2.9 – 11.8 −16.6−10.4, *p <* 0.05			Muscle activation bursts were enhanced on the paretic side while decreased on the non-paretic side. Variability of muscle activation and EMG activity during swing were diminished on the paretic side.
	3x over 5-week period								
Thaut et al. (35)	**I1:** CT gait with RAS		**I2:** CT gait without RAS		Walking	**Mean change of coefficient of variation (%) of the gastrocnemius:** With RAS: 69 (11)/without RAS: 33 (31) Time x group: MW_calc_ = 138, *p <* 0.02			RAS enhances more regular motor unit recruitment patterns.
	Twice a day, 30 min each, 5x/week, 6 weeks								
Thaut et al. (36)	**I1:** With auditory cue		**I2:** Without auditory cue		Target reaching	Rhythm–no rhythm: * Arm movement time (ms): 1,425 (185) – 1,446 (289), *p >* 0.05 * Arm acceleration (% deviation): 38.7 (8.05) – 168.2 (36.1), *t =* 19.1, *p <* 0.001 * Arm coefficient of variation (%): 13 – 20, *t =* 3.205, *p =* 0.013 * Wrist trajectory variability (%): decrease of 40.5 – 26.1%, t: 2 = 411, *p =* 0.042 * Elbow ROM (°): 31.47 (9.65) – 28.19 (9.96), *t =* 3.44, *p =* 0.007 * Mean shoulder displacements (cm): 18.7 – 16.8, *p >* 0.05			The immediate benefit of rhythmic cuing on arm control provides a strong rationale to apply rhythmic entrainment to the recovery of arm function in long-term hemiparetic stroke rehab.

*I1–4, interventions 1–4; RAS, rhythmic auditory stimulation; Ext, extension; FCL, flexor carpi ulnaris; co-act, co-activation; Hz, Hertz; ØA_i_, movement amplitude arms of involved side; ØA_ni_, movement amplitude arms of non-involved side; ØH_i_, movement amplitude hips of involved side; ØH_ni_, movement amplitude hips of non-involved side; MPA_i_L_i_, mean point estimates of relative phase between arms and legs of involved side; MPA_ni_L_ni_, mean point estimates of relative phase between arms and legs of non-involved side; RPI_ni_, relative power index non-involved side; ØP, movement amplitude pelvis; ØT, movement amplitude thorax; ØPT, movement amplitude pelvis/thorax; MPPT, mean point estimates of pelvis and thorax; μV, microvolt; °, degrees; ms, milliseconds; MU, motor units; GMi, ipsilateral gluteus medius; GMc, contralateral gluteus medius; p, paretic; np, non-paretic; PPC, posterior parietal cortex; h, hour; cm, centimeter; x, times; hor, horizontal; vert, vertical; NDT, neurodevelopmental treatment (Bobath); CT, conventional therapy; vs., versus; >, greater than; <, smaller than; = , equal to; PIP, proximal interphalangeal joint; MEP, motor-evoked potential; CM, central motor; GDI, gait deviation index; MW_calc_ = Mann–Whitney calculations; LMI, locomotor imagery; ANOVA-RM, analysis of variance - repeated measures; UE, upper extremity; DF, dorsal flexion; IC, initial contact*.

For the analysis, the percentage of change between SBI and no-sound conditions was examined. Data figures can be found as Appendices S3–S5. To facilitate the qualitative interpretation of the RCTs, forest plots were created using RevMan 5.3 (37). The number of participants, mean differences, and standard deviations were inserted in the RevMan 5.3 template. When the necessary data were not available, authors were contacted to complete the data form. If authors did not respond, missing data were manually calculated using the RevMan 5.3 calculator, if possible. To calculate pooled effect sizes, inverse variance was used as statistical method, a random-effects model was used as an analysis model, and standardized mean differences (SMDs) were calculated as the effect measure. Heterogeneity between the studies was assessed using *I*^2^ statistics, together with magnitude and direction of effects for overlapping ranges (38, 39). Cochrane guidelines were used to interpret the heterogeneity: 0–40% (might not be important), 30–60% (may represent moderate heterogeneity), 50–90% (may represent substantial heterogeneity), and 75–100% (considerable heterogeneity) (39). Effect sizes were categorized as a standard mean effect size of 0 representing no change, 0.2 representing a small effect, 0.5 representing a medium effect, and 0.8 representing a large effect (40). Confidence intervals (CIs) were set to 95%.

## Results

### Study Selection

**Figure 1:**
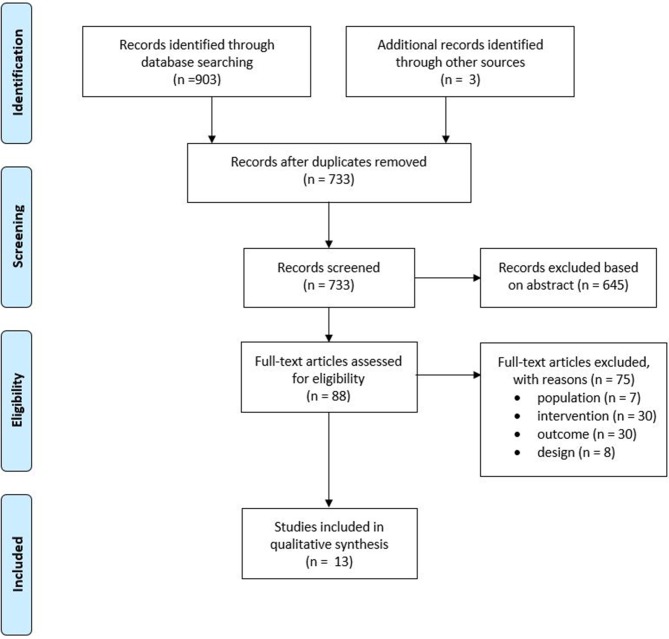
Preferred Reporting Items for Systematic Review and Meta-Analysis Statement (PRISMA) flowchart of the included studies.

Of the 733 unique studies obtained from all databases, 13 studies met all inclusion criteria. The study selection process is depicted in Figure 1. Concerning the quality assessment (see Table 3), the nine cross-sectional studies had a median score of seven, with a maximum score of eight and a minimum of four. In total, five studies had a good methodological quality, while the others were of fair to poor quality. The majority of studies used a selected group of subjects who did not represent the target population, and no study justified their sample size. Concerning the quality assessment of the RCTs, a median score of five was observed, with a maximum of nine and a minimum of five (see Table 4). Most studies did not meet the criteria of blinding the subjects and therapist, as this does not seem possible with respect to treatment. It would be very difficult to blind people as to receiving or executing SBI compared to a placebo or no therapy.

**Table 3:** Risk of bias for cross-sectional studies with the Newcastle–Ottawa Quality Assessment Scale.

**Author**	**Selection (/4)**				**Comparability (/2)**	**Outcome (/3)**		**Total NOS**
	**1**	**2**	**3**	**4**	**5**	**6**	**7**	
Aluru et al. (24)	★		★	★	★★	★★	★	8/9
Ford et al. (25)			★			★★	★	4/9
Kim et al. (27)	★		★	★	★	★★	★	7/9
Malcolm et al. (29)			★	★		★★	★	5/9
Prassas et al. (30)			★	★		★★	★	5/9
Sethi et al. (31)			★	★★	★	★★	★	7/9
Shin et al. (33)	★		★	★	★★	★★	★	8/9
Thaut et al. (34)			★	★		★★	★	5/9
Thaut et al. (36)	★		★	★★	★	★★	★	8/9

*1, representativeness of the sample; 2, sample size; 3, non-respondents; 4, ascertainment of the exposure tool; 5, subjects are comparable/confounding; 6, assessment of outcome; 7, statistical test; NOS, Newcastle–Ottawa scale; LOE, level of evidence*.

**Table 4:** Risk of bias of randomized controlled trials with the Physiotherapy Evidence Database (PEDro) scale.

**Author (Year)**	**Eligibility**	**Random allocation**	**Concealed allocation**	**Baseline comparability**	**Blinding subjects**	**Blinding therapist**	**Blinding assessors**	**Adequate follow-up**	**Intention-to-treat analysis**	**Between-group statistical comparison**	**Point measures and measures of variability**	**Total PEDro score**
Kim et al. (26)	1	1	0	1	0	0	0	0	1	1	1	5/10
Mainka et al. (28)	1	1	1	1	0	0	1	0	0	1	1	6/10
Shahine and Shafshak (32)	1	1	1	1	1	0	1	1	1	1	1	9/10
Thaut et al. (35)	0	1	0	1	0	0	0	0	1	1	1	5/10

### Study Characteristics

In total, data from 279 stroke survivors (98 females, 174 males) were included in this study. The examined participants had a mean age of 61 years (range: 44–73 years) and an average time post-stroke of 24 months (range: 16.1 days−5.5 years) (24–36). One study did not provide mean age and time post-stroke of their included population (25).

Four studies were RCTs investigating the effectiveness of SBI by comparing motor activities with and without sound (26, 28, 32, 35). Additionally, two studies examined the effectiveness of SBI by means of a pre/post-design (29, 33), and seven studies investigated the immediate effects of SBI on motor tasks in a cross-sectional study (24, 25, 27, 30, 31, 34, 36).

Motor tasks included both upper and lower limb tasks and varied across studies. Upper limb tasks were generally related to reaching exercises (27, 29, 31, 32, 36), but one study investigated wrist flexion/extension (24). Lower limb tasks mostly consisted of walking trials which could be performed over ground or on a treadmill (25, 26, 30, 33–35). One study examined standing balance in a static condition (28).

The majority of studies generated rhythmic sound sequences via a metronome (25–27, 29, 31, 36), two used an actual or a software package of a synthesizer/keyboard (28, 30), and two others a combination of both (33, 35). In these studies, a baseline assessment was performed to calculate the reach or step frequency. Afterward, an increased or decreased auditory frequency was provided to examine the influence of the rhythmic sounds' characteristics on the movement. These rhythmic sound sequences were generated via individual sounds generated by synthesizers or metronomes. In the study by Aluru et al. (24), patients were exposed to four different types of sounds: metronome sounds, a baby's laughter, self-selected music, and silence. Only one study used actual music—Thaut et al. (34) used a music piece of renaissance dance style orchestrated for woodwinds, harpsichord, and percussion. One study did not specify the manner of sound production or apparatus used yet elaborately described the motor task procedure, time of execution, and sound frequency (0.25–1.0/s) (32). The outcome measures and motion capture systems used to assess motor behavior can be found in Table 1.

### Synthesis of Results

#### Research Question 1: Is Motor Behavior Altered During SBI?

The immediate effect of SBI on muscle activity was examined by three studies (24, 27, 34), on joint angles by four (24, 27, 30, 36), and temporal parameters during reaching by three studies (25, 31, 36) (see Appendix S3).

During SBI, significant differences in gastrocnemius muscle activity were found during walking compared to no sound, a 10.8% increase in amplitude at push-off, a 16.5% increase in amplitude variation, and a 10.4% decrease in amplitude during swing (34). Concerning upper limb muscle activity, a non-significant increase in the maximum voluntary isometric contraction of biceps activity of 0.34% and a significant increase in triceps activity of 18.18% were observed (27). Moreover, a decrease of 10% was found in the co-contraction ratio of the aforementioned muscles during reaching (27). Aluru et al. (24) investigated the changes of SBI between three types of stroke patients: spastic paresis, spastic co-contraction, and minimal paresis. They concluded that listening to a metronome or self-selected songs increased wrist flexor activity in patients with spastic paresis but not those with spastic co-contraction. For patients with minimal paresis, results were unclear (24).

During walking, the vertical displacements of the center of mass significantly decreased with 15.15% between SBI and the no-sound condition (30). However, no significant differences were found concerning range of motion (RoM) of the hip and knee, trunk, and pelvic tilt angle (30). Aluru et al. (24) concluded that patients with spastic paresis improved their wrist extension with a metronome during reaching, while the spastic co-contraction group did improve the amplitude of their wrist extension without cue. On the other hand, the minimal paresis group improved when listening to “happy” sounds (24).

Temporal parameters were only assessed during reaching. Studies found that the deviation of the optimal peak acceleration decreased by 76% (36) and peak velocity increased by 34% in the sound condition (31), while movement time did not significantly differ between the two conditions (36). Increased coordination of the arm/leg and trunk during walking (25) and decreased variability (range: 12.5–214%) of the upper limb during reaching (31, 36) were observed during SBI.

In summary, there is a small amount of evidence that after SBI, muscle activity of the gastrocnemius and RoM of the upper limb increased, while also normalizing acceleration, enhancing peak velocity, and decreasing variability.

#### Research Question 2: Is Motor Behavior Altered After SBI?

Only two studies examined the effects of SBI on motor behavior after a period of 10–28 days of therapy (29, 33) (see Appendix S4), and both examined different motor tasks that necessitated different outcome measures, which makes the comparison difficult. During reaching tasks, the segmental contribution of the trunk seemed to decrease, while the contribution of shoulder flexion increased (29). No significant differences were found pre- and post-SBI concerning the contribution of elbow extension (29). In addition, movement time decreased by 23%, while reach velocity increased by 20% (29). Of the 28 joint angle parameters during walking assessed by Shin et al. (33), only two were significant before and after SBI in stroke patients. Maximal knee flexion during mid-swing and maximal dorsiflexion during terminal stance increased by 13 and 17%, approximately (33).

In summary, no consensus could be reached, due to the variety of outcome measures and motor tasks. However, there was a tendency for SBI to affect several biomechanical parameters.

#### Research Question 3: Is SBI Effective in Improving Motor Behavior?

**Figure 2:**
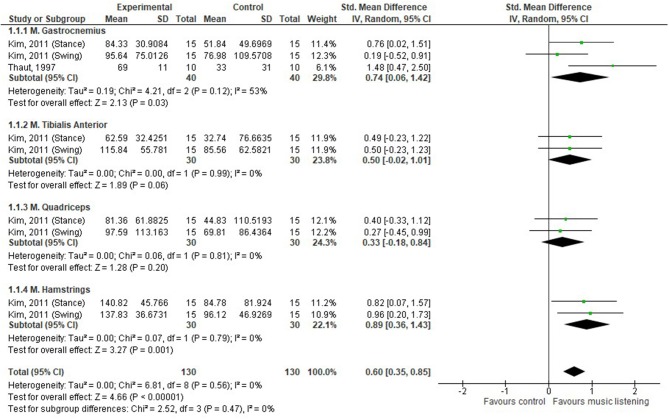
Effectiveness of music listening on muscle activity.

In order to examine whether the observed effects in the pre/post-designs are due to SBI or natural recovery, results should be compared to a control group, which was the case for four studies (26, 28, 32, 35). Our analysis shows that there was a moderate effect of music listening on muscle activity (SMD 0.60, 95% CI 0.35–0.85) (26, 35), as depicted in Figure 2. The gastrocnemius muscle was the only one assessed in both studies, and a moderate effect was seen in favor of SBI (SMD 0.74, 95% CI 0.06–1.42). However, a considerable amount of heterogeneity was observed (*I*^2^ = 53%). No significant differences were observed for the tibialis anterior muscle and quadriceps muscle, while a large effect was observed for the hamstring muscles, SMD 0.89 (95% CI 0.36–1.43). Concerning joint angles during walking, SBI seemed to improve RoM of the knee by 25 and 39% in the control and the experimental group, respectively (26). No significant differences were found between treadmill training with and without RAS in static balance outcome measures as evaluated by means of center of pressure (COP)–based measures (28). Finally, Shahine and Shafshak's (32) was the only study looking at upper limb activities; bilateral arm training with SBI significantly increased central motor conduction time (which is the difference between the peripheral and cortical latency of the signal), motor-evoked potential resting threshold, and amplitude ratio compared to the control group (see Appendix S5).

In summary, there is a limited amount of evidence that SBI has a positive effect on motor behavior—muscle activity of the gastrocnemius and hamstring muscles improved, in addition to neurophysiological parameters and knee flexion amplitude.

## Discussion

### Do Sound-Based Interventions Influence Motor Behavior?

Although recent literature concluded that music interventions are beneficial for improving clinical outcome measures of both upper and lower extremities after stroke (3, 4, 6), little research has been performed concerning the underlying mechanisms of SBI. Although biomechanical analysis cannot fully distinguish between true recovery and compensation, as neural mechanisms should also be investigated (e.g., motor control), it is a first step in understanding and explaining the observed clinical improvements. We reviewed a total of 12 studies that provided biomechanical data obtained from 240 survivors during the execution of a variety of motor tasks. The overall risk of bias of the included studies was moderate; 10 studies were of good quality, while eight were of fair to poor quality. However, the great heterogeneity between studies, mainly due to the great variation in outcome measures and assessment at different time points, made comparison difficult.

1. Is motor behavior altered *during* SBI?—Yes!
  High-quality studies all reported significant improvements in movement quality, e.g., decreased co-contraction, increased peak velocity, and decreased variability. So it seems that SBI might be able to improve movement control.
2. Is motor behavior altered *after* SBI?—Not clear…
  Although two studies (each examining a different motor task) showed that several biomechanical parameters were improved after SBI, no clear conclusions could be made. However, there is a tendency for SBI to aid motor behavior.
3. Is SBI *effective* in improving motor behavior?—Yes, but more evidence is needed.
  Studies examining the lower limbs showed signs of normalization of muscular activity and motor recruitment (26, 35), while no improvements were observed concerning static balance (28). Furthermore, only one study investigated upper limb movements (32). Therefore, more qualitative research is needed.

### How Do Sound-Based Interventions Influence Walking and Reaching Performance?

1. The effect of SBI on walking

After stroke, problems with foot clearance are commonly reported, resulting in an increased fall risk (41–43). SBI seems to be able to improve clearance in swing by increasing knee flexion and push-off activity, which is beneficial during gait rehabilitation after stroke to decrease tripping and enhance walking speed. An effective push-off is important for leg swing acceleration and knee flexion (44, 45). These improvements might be one of the underlying causes to explain the significant changes seen in walking speed, stride length, gait cadence, and stride symmetry after music interventions in stroke patients (3). It seems that some form of true recovery might have taken place at the level of the knee; however, too little research has been performed in order to fully explain the mechanisms, as we still have no idea what drives this recovery process based on the available literature.

1. The effect of SBI on reaching tasks

Although improvements in motor control, cortical reorganization, and/or increased central excitability were observed during reaching, too little qualitative research was performed on kinematic parameters after SBI. It is suggested that MLI aids in motor planning activities, as listening to music engages a complex network of brain regions, in both the auditory and the motor system (46). To date, evidence is still too limited to conclude that SBI is able to induce true recovery after stroke. In addition, studies concerning neuroplasticity suggest that the first weeks after a stroke are crucial for inducing functional and structural cortical reorganization (47, 48), whereas the majority of studies assessed chronic stroke patients. Therefore, future research should include sub-acute stroke patients since cortical reorganization is more apparent in this population, which is a sign of true recovery. To date, no research has been performed on the effect of SBI during reaching in sub-acute stroke patients.

### Music or Rhythmic Sound Sequences?—Directions for Future Research

Only two studies used actual music pieces or musical instruments as part of the intervention (24, 34). The majority of studies used a metronome or a synthesizer to play a certain rhythm. However, walking is a more dynamic situation which cannot be entirely explained as consecutive beats (49). Music, on the other hand, has a more complex auditory stream of rhythmic, dynamic, harmonic, and timbral structures. These different parameters can map different gait events, not just synchronizing heel contact to a beat, as exemplified by Rodger and Craig (49): “a patient may either lift his/her toe off with a beat, place the heel with a chord, or swing the leg with part of a melody, and still have the veridical experience of being in time with the sound”. Studies have already shown that moving to music compared to the sound of a metronome resulted in a faster walking speed and decreased synchronization errors (50–52). Furthermore, listening to music activates cortical and paralimbic areas related to neural systems of reward and emotion (53), which makes music an intervention that can be rewarding and motivating and at the same time regulate emotions, arousal, and cognitive functions (54), especially when patients are able to choose the music themselves (53, 55). When healthy volunteers listen to self-selected music, increased muscle activity and heart and respiration rate were observed compared to non-self-selected music (53). Even though the benefits of MLI are well-described in healthy adults, the lack of studies using actual music pieces in this review highlights the need for future research on MLIs.

## Limitations

There are a few limitations of this review that should be acknowledged. First, during the systematic literature search, only studies written in English, Dutch, German, or French were included. It is therefore possible that relevant studies and important information were missed during the search process. Second, some caution with these findings is required since conclusions were sometimes based on the results of a single study. The heterogeneity in outcome measures made it difficult to find comparable results. Third, although clinical improvements have been reported repeatedly, the amount of research regarding the quality of the movement after SBI is very limiting.

## Conclusion

There is evidence concluding that SBI is able to induce some form of true recovery during walking after stroke, while it was difficult to provide evidence for reaching tasks. There was a great amount of heterogeneity between the included studies, hampering clear conclusions. At this point, it is still unknown what the underlying mechanisms of the observed improvements are. Several important gaps in the literature were determined, which necessitates further qualitative examination. Future research should include larger study samples, sub-acute stroke patients, and actual music pieces instead of only rhythmical sounds to examine music interventions after stroke.

## Data Availability Statement

Publicly available datasets were analyzed in this study. All datasets analyzed for this study are included in the article/Supplementary Material.

## Author Contributions

TV co-designed the study, carried out the screening procedure, performed the risk-of-bias assessment, was involved with data extraction and interpretation of data, and drafted the manuscript. KD'A co-designed the study, took part in the screening procedure, performed the risk-of-bias assessment, and was involved in data interpretation and drafting of the manuscript. JO'B took part in the screening procedure and risk-of-bias assessment and was involved with data interpretation and drafting of the manuscript. EC co-designed the study, carried out the screening procedure, and was involved in data interpretation and drafting of the manuscript. All authors gave final approval for publication.

### Conflict of Interest

The authors declare that the research was conducted in the absence of any commercial or financial relationships that could be construed as a potential conflict of interest. The reviewer AD and handling Editor declared their shared affiliation at the time of review.
