# Supplementary material for: The Influence of Sound-Based Interventions on Motor Behavior After Stroke: A Systematic Review
Source: Front Neurol. 2019 Nov 1;10:1141. doi: 10.3389/fneur.2019.01141 (PMC6838207; doi:10.3389/fneur.2019.01141)
Supplement: Supplementary file 2 [file Table_2.DOCX]

| **Database** | **Search strategy** |
| --- | --- |
| Web of Science and Pubmed/Medline | (music OR "music supported therapy" OR rhythm OR "rhythmic auditory stimulation" OR rhythmic OR "acoustic") AND (muscle OR electromyography or "peripheral physiology" or kinematics or biomechanics) AND (stroke OR hemiplegia OR "cerebrovascular disorders" OR "cerebrovascular accident") |
| PEDro | Music AND stroke |
| Cochrane Library | Music OR RAS AND stroke |
| **Appendix S2.** Search Strategy | |
