# Supplementary material for: The Influence of Sound-Based Interventions on Motor Behavior After Stroke: A Systematic Review
Source: Front Neurol. 2019 Nov 1;10:1141. doi: 10.3389/fneur.2019.01141 (PMC6838207; doi:10.3389/fneur.2019.01141)
Supplement: Supplementary file 3 [file Table_3.DOCX]

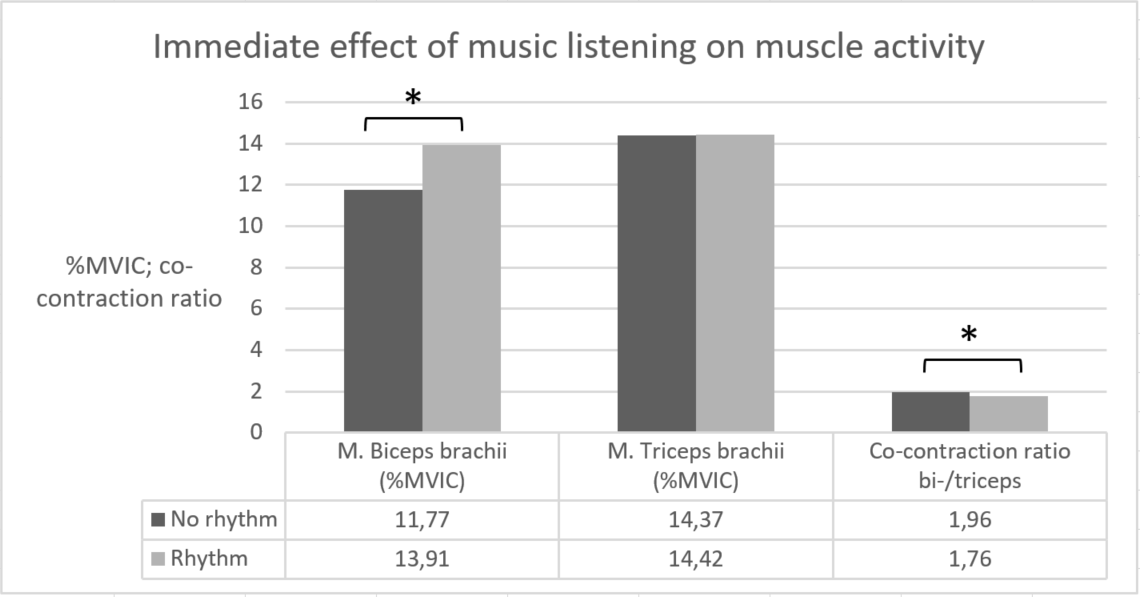

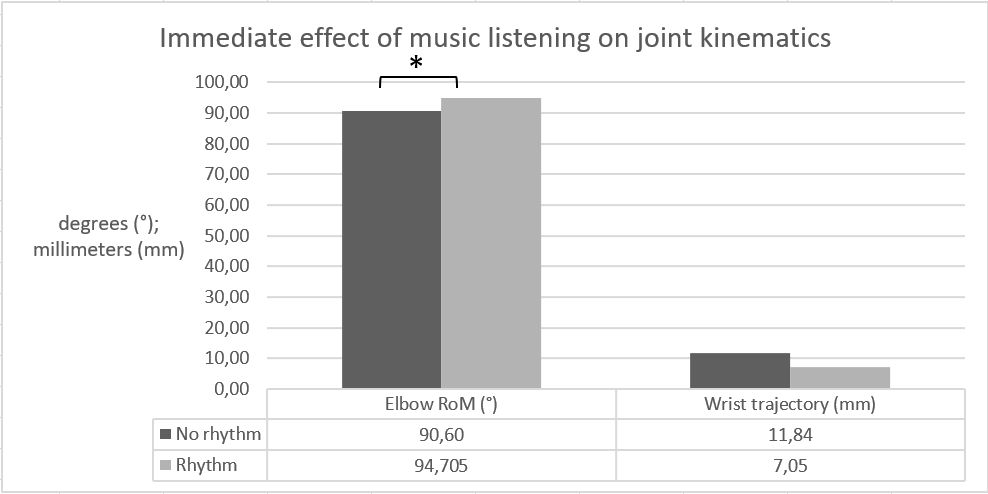


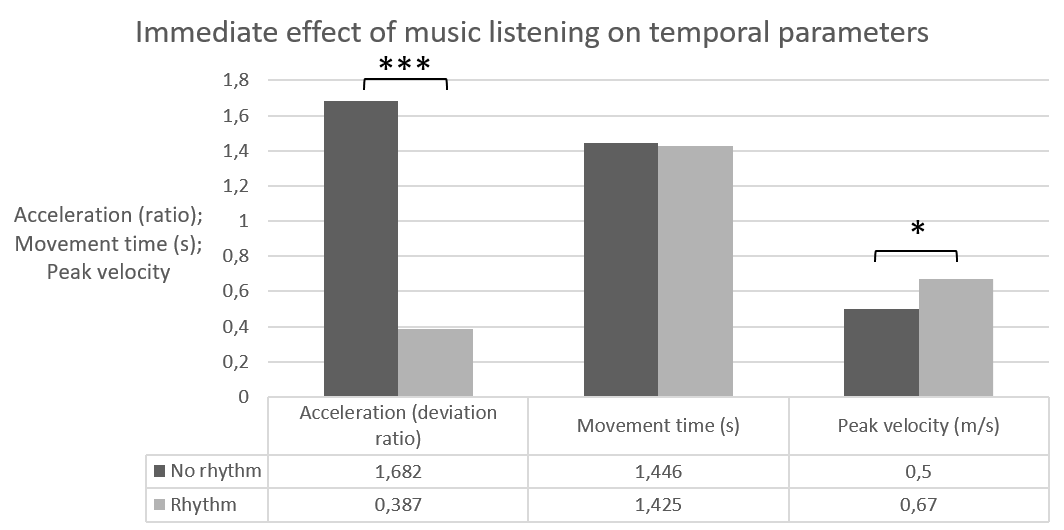

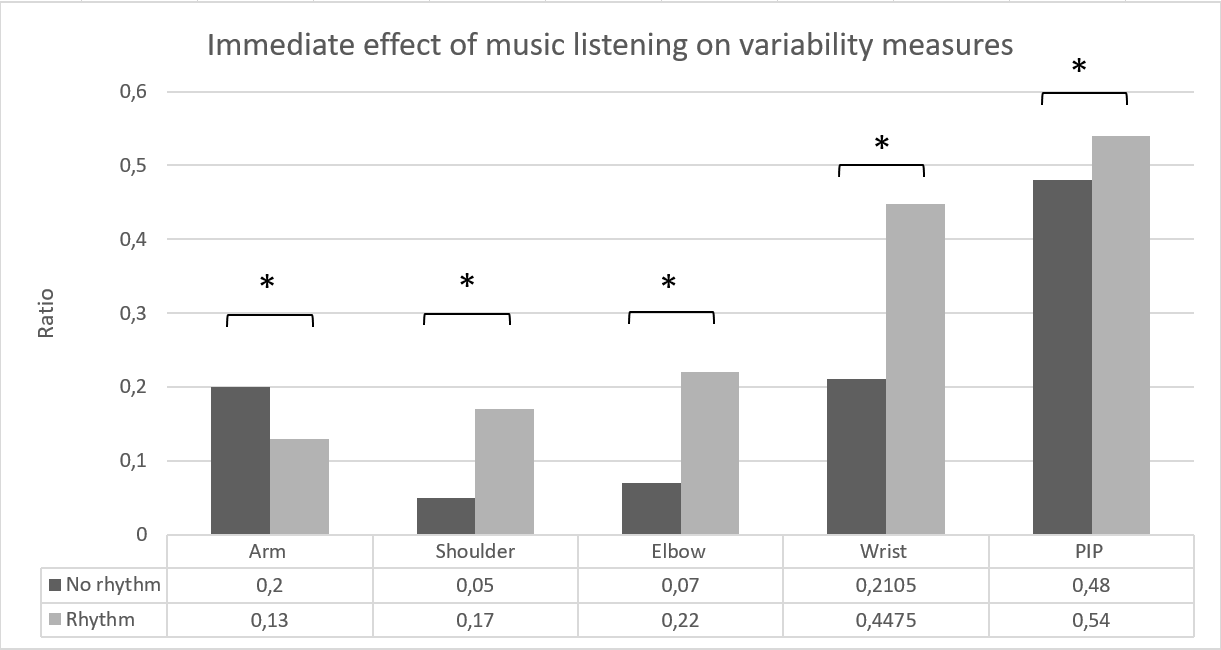


**Appendix S3.** Changed motor behaviour during music listening

%: percentage, MVIC: maximal voluntary isometric contraction, s: seconds, m: meter, PIP: proximal interphalangeal joint

Comparison t-test: *** p<0.001, *p<0.05
