# Supplementary material for: The Influence of Sound-Based Interventions on Motor Behavior After Stroke: A Systematic Review
Source: Front Neurol. 2019 Nov 1;10:1141. doi: 10.3389/fneur.2019.01141 (PMC6838207; doi:10.3389/fneur.2019.01141)
Supplement: Supplementary file 4 [file Table_4.DOCX]

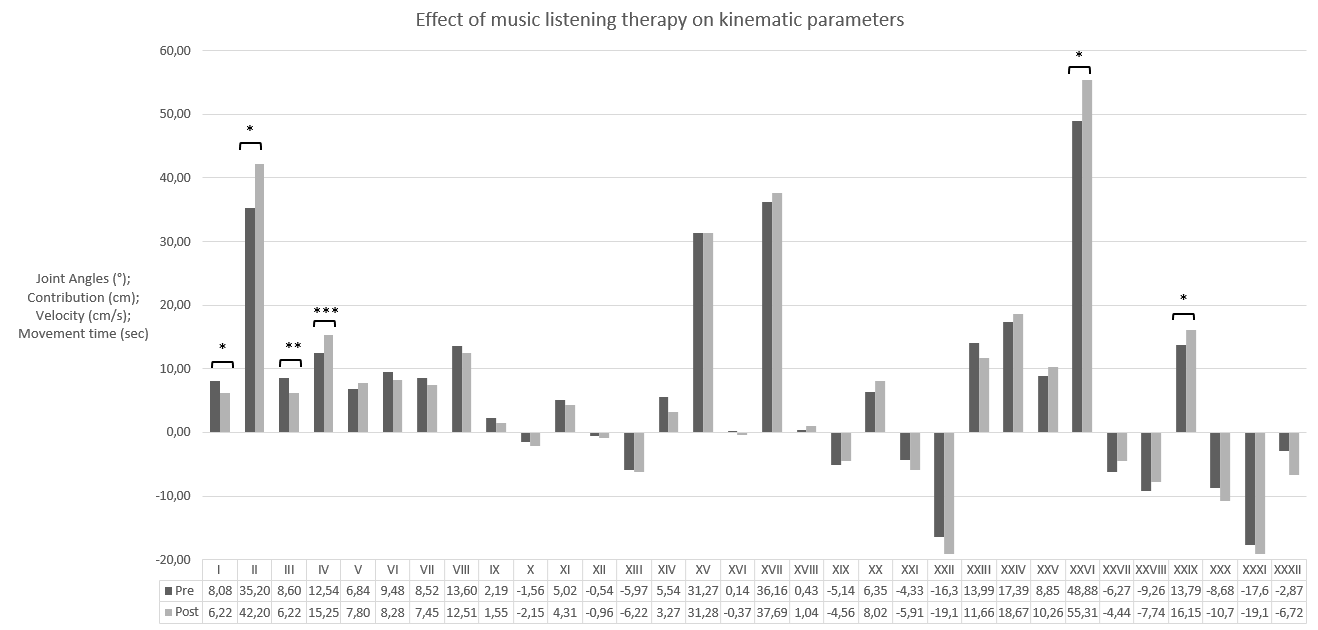


**Appendix S4.** Changed motor behaviour after music listening

**I: Movement time** (s: seconds), **II: Reach velocity** (cm/s: centimetre per seconds), **III: Trunk contributions** (cm), **IV: Shoulder contribution** (cm), V: Elbow (cm), VI: Pelvic tilt at initial contact (°: degrees), VII: Minimal pelvic tilt (°), VIII: Maximal pelvic tilt (°), IX: Pelvic obliquity at initial contact, X: Minimal pelvic obliquity (°), XI: Maximal pelvic obliquity (°), XII: Pelvic rotation at initial contact (°), XIII: Minimal pelvic rotation (°), XIV: Maximal pelvic rotation (°), XV: Hip flexion at initial contact (°), XVI: Minimal hip flexion at push off (°), XVII: Maximal hip flexion terminal swing (°), XVIII: Hip abduction at initial contact (°), XIX: Maximal hip abduction mid swing (°), XX: Maximal hip adduction mid stance (°), XXI: Hip rotation at initial contact (°), XXII: Maximal external hip rotation mid swing (°), XXIII: Maximal internal hip rotation stance (°), XXIV: Knee flexion at initial contact (°), XXV: Minimal knee flexion terminal stance (°), **XXVI: Maximal knee flexion mid swing** (°), XXVII: Ankle dorsiflexion at initial contact (°), XXVIII: Maximal ankle plantar flexion at push off (°), **XXIX: Maximal ankle dorsiflexion terminal stance** (°), XXX: Foot rotation at initial contact (°), XXXI: Maximal external foot rotation mid stance (°), XXXII: Maximal internal foot rotation at push off. *p<0.05, **p<0.01, ***p<0.001
