# Supplementary material for: The Influence of Sound-Based Interventions on Motor Behavior After Stroke: A Systematic Review
Source: Front Neurol. 2019 Nov 1;10:1141. doi: 10.3389/fneur.2019.01141 (PMC6838207; doi:10.3389/fneur.2019.01141)
Supplement: Supplementary file 5 [file Table_5.DOCX]

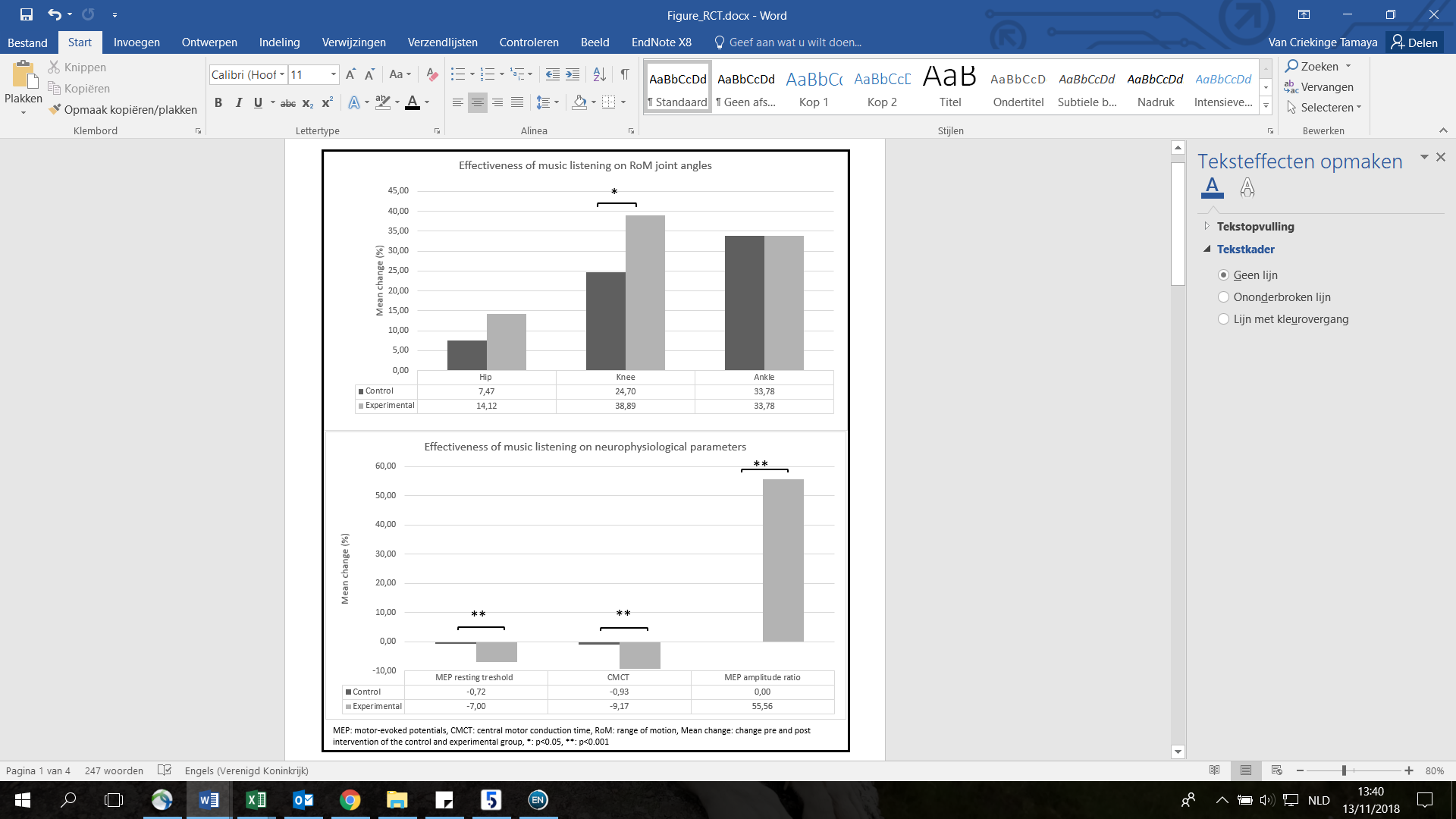

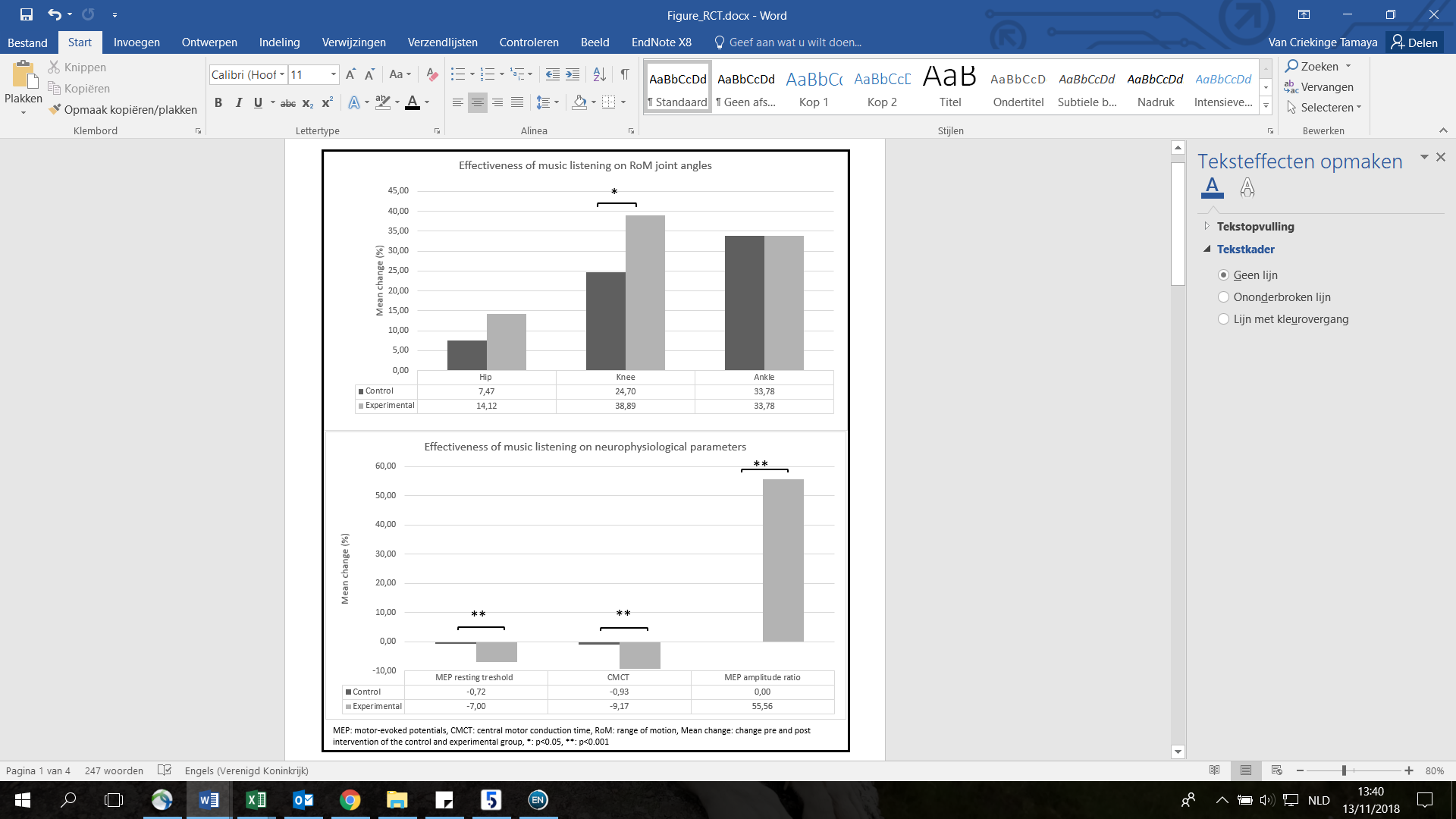


**Appendix S5.** Effectiveness of music listening on motor behaviour

%: percentage, MEP: motor-evoked potentials, CMCT: central motor conduction time, RoM: range of motion. *p<0.05, **p<0.001
